# Perinatal Natural History of the Ts1Cje Mouse Model of Down Syndrome: Growth Restriction, Early Mortality, Heart Defects, and Delayed Development

**DOI:** 10.1371/journal.pone.0168009

**Published:** 2016-12-08

**Authors:** Millie A. Ferrés, Diana W. Bianchi, Ashley E. Siegel, Roderick T. Bronson, Gordon S. Huggins, Faycal Guedj

**Affiliations:** 1 Mother Infant Research Institute (MIRI) at Tufts Medical Center and Floating Hospital for Children, Boston, MA, United States; 2 Department of Obstetrics and Gynecology, Division of Maternal Fetal Medicine, Beth Israel Deaconess Medical Center, Boston, MA, United States; 3 Rodent Histopathology Core, Dana-Farber/Harvard Cancer Center, Boston, MA, United States; 4 Molecular Cardiology Research Institute (MCRI) at Tufts Medical Center, Boston, MA, United States; IGBMC/ICS, FRANCE

## Abstract

**Background:**

The Ts1Cje model of Down syndrome is of particular interest for perinatal studies because affected males are fertile. This permits affected pups to be carried in wild-type females, which is similar to human pregnancies. Here we describe the early natural history and growth profiles of Ts1Cje embryos and neonates and determine if heart defects are present in this strain.

**Methods:**

Pups were studied either on embryonic (E) day 15.5, or from postnatal (P) day 3 through weaning on P21. PCR amplification targeting the *neomycin* cassette (present in Ts1Cje) and *Sry* (present in males) was used to analyze pup genotypes and sex ratios. Body weights and lengths, as well as developmental milestones, were recorded in Ts1Cje mice and compared to their wild-type (WT) littermates. Histological evaluations were performed at E15.5 to investigate the presence or absence of heart defects. Pups were divided into two groups: Ts1Cje-I pups survived past weaning and Ts1Cje-II pups died at some point before P21.

**Results:**

Ts1Cje mouse embryos showed expected Mendelian ratios (45.8%, n = 66 for Ts1Cje embryos; 54.2%, n = 78 for WT embryos). Histological analysis revealed the presence of ventricular septal defects (VSDs) in 21% of Ts1Cje E15.5 embryos. After weaning, only 28.2% of pups were Ts1Cje (185 Ts1Cje out of 656 total pups generated), with males predominating (male:female ratio of 1.4:1). Among the recovered dead pups (n = 207), Ts1Cje (63.3%, n = 131, *p*<0.01) genotype was found significantly more often than WT (36.7%, n = 76). Retrospective analysis of Ts1Cje-II (pre-weaning deceased) pups showed that they were growth restricted compared to Ts1Cje-I pups (post-weaning survivors). Growth restriction correlated with statistically significant delays in achieving several neonatal milestones between P3 and P21 compared to Ts1Cje-I (post-weaning survivors) neonates and WT littermates.

**Conclusions:**

Ts1Cje genotype is not associated with increased early *in utero* mortality. Cardiac defects, specifically VSDs, are part of the phenotype in this strain. There is increased neonatal mortality in Ts1Cje pups, with sex differences observed. Ts1Cje mice that died in the neonatal period were more likely to be growth restricted and delayed in achieving neonatal developmental milestones.

## Introduction

Down syndrome (DS) is the most frequent genetic cause of intellectual disability. DS affects 8.3 to 14.3 in 10,000 live births with a male predominance [1–3]. The clinical findings in DS are complex, and include cognitive delays, motor deficits, cardiac malformations, and early-onset Alzheimer’s disease [4].

The most well-studied mouse models that are partially trisomic for genes orthologous to human chromosome 21 (*Hsa21)* include Dp(16)1Yey/+ (hereafter called Dp16), Ts65Dn and Ts1Cje. These models demonstrate many phenotypic changes comparable to those in DS. They represent an important tool by which to investigate the molecular and cellular origins of the phenotype and to evaluate the effects of different treatments [5–7]. Ts65Dn mice, which are trisomic for 128 Hsa21 orthologous protein and non-protein coding genes, are the best-studied model of DS. These mice have a well-characterized phenotype [6,8]. Ts65Dn males are, however, generally infertile, and Ts65Dn females have poor reproductive outcomes [9]. Moore *et al* (2010) identified occasional fertile Ts65Dn males, however, there are still gaps in knowledge regarding the long-term sustainability and reproductive performance of these males [10]. The phenotype of the more recently generated Dp16 strain (trisomic for 145 protein and non-protein coding genes) is less well described [7,11] and, in contrast with the Ts65Dn mouse model, does not show abnormal embryonic forebrain development and early neonatal developmental milestones [12].

The Ts1Cje mouse model contains a reciprocal translocation with a smaller trisomic region (77 genes) than Ts65Dn and a monosomy of the distal part of chromosome 12 (7 genes), but it has some comparable, although milder, behavioral and cognitive deficits [7, 8, 13–15]. In contrast to Ts65Dn, Ts1Cje males are fertile [7]. This allows the transmission of the derivative chromosome through the paternal germ line and avoids the confounding factor of maternal trisomy on fetal development *in utero*.

To date, studies of Ts1Cje mice have focused mainly on the adult brain phenotype and cognitive deficits [5, 14]. We have previously published data on the embryonic brain transcriptome and neonatal behavioral abnormalities in Ts1Cje mice [15]. In the course of performing the prior experiments we noted neonatal deaths and wondered whether these were associated with the Ts1Cje genotype?

Here we generated a large cohort of embryos and neonates to analyze the perinatal natural history of the Ts1Cje mouse model as a function of pup sex and postnatal growth patterns, and to assess for the presence or absence of congenital heart defects. Studies in other mouse models have shown the presence of congenital heart defects in 8–15% of Ts65Dn pups [16, 17] and 37% of Dp16 embryos [5, 18]. At the time we began the cardiac part of the study (2013), it was unknown whether the Ts1Cje mice exhibited congenital heart defects.

## Materials and Methods

### Animal Housing and Breeding

This study was carried out in strict accordance with the recommendations in the Guide for the Care and Use of Laboratory Animals of the National Institutes of Health. All experimental procedures were approved by the Institutional Animal Care and Use Committee (IACUC) of Tufts University [Protocols B2013-20 and B2015-171]. All surgery was performed using 2.5% isoflurane in a 3/7 0_2_/N_2_O mixture anesthesia and euthanized by decapitation, and all efforts were made to minimize suffering. Animal health was monitored on a daily basis and mice presenting skin lesions, inactivity, change in eating or drinking habits, ≥15% weight loss, piloerection or roughening of fur, hunched posture were euthanized using CO_2_ according to Tufts University guidelines. Ts1Cje males were crossed with C57Bl/6J females for six generations before experiments started. Mice were housed in standard cages with food and water *ad libitum* and under a controlled environment (temperature = 20°C; humidity = 60%; light/dark cycle of 12 hours). Both embryos and offspring resulting from these matings were used.

### Embryonic Day 15.5 Studies

For embryonic studies, matings (one Ts1Cje male and two C57Bl/6J females) were set up every day between 4:00 and 5:00 PM and separated every morning (8:00–9:00 AM) after examination for the presence of vaginal plugs. The presence of the vaginal plug was defined as embryonic day 0.5 (E0.5). The pregnancy was confirmed by a 15–20% weight gain 10 days later [19].

At embryonic day 15.5 (E15.5), pregnant females were anesthetized with 2.5% isoflurane in a 3/7 0_2_/N_2_O mixture and euthanized by decapitation. Embryos were extracted in ice-cold phosphate-buffered-saline (PBS 1X, pH 7.4) and Theiler staging (http://www.emouseatlas.org/emap/ema/theiler_stages/theiler_stages.html) was used to confirm their gestational age. A tail snip was removed from each embryo (n = 144 embryos) and stored at -20°C for genotyping. Embryonic trunks were preserved in Bouin’s fixative solution (Rowley Bio, Danvers, MA) for further histological analyses.

### Ts1Cje Mouse Colony Registry and Natural History Studies

Starting in February 2012, a registry was created to record detailed information about Ts1Cje breeding rates, litter sizes, dates of birth, pup sex, and genotypes. This information was used to analyze the genotypes (Ts1Cje vs. WT) and sex ratios of all pups that survived after P21 between March 2012 and December 2015. A total of 656 pups from 131 different litters were analyzed. To further investigate neonatal mortality, we recovered the maximum number of dead pups or their remainders (n = 207) during the period from 05/01/13 to 12/30/15. Pregnant females and delivered litters were checked twice every day (9:00 AM and 5:00 PM). For each dead pup, a tail snip was stored at -20°C for subsequent genotyping.

### DNA Extraction From Tail Snips and Ear Punch Biopsies

Tail snips from embryos and dead neonates were incubated with 500 μl of TSE lysis buffer (50 mM Tris pH = 7.5; 0.5% SDS and 20 mM EDTA) containing 0.7 mg/ml of proteinase K (Qiagen, Catalog N: 19133) at 55°C overnight on a thermomixer (Eppendorf, Hauppauge, NY). Following the digestion step, DNA was purified using the isopropanol precipitation protocol described previously [20]. DNA concentrations were measured as absorbance at 260 nm on the Nanodrop instrument (Thermo Fisher Scientific, Waltham, MA). For pups that survived, ear punches were obtained for genotyping. The same protocol as for tail snips was used, but with half the volume of reagents.

### Analysis of Genotype and Sex Distribution in Embryos and Neonates Using Multiplex Polymerase Chain Reaction Amplification

To determine the natural history of the Ts1Cje mice, we analyzed genotype and sex ratios in three different groups: 1) embryos at day E15.5 (embryonic life); 2) dead pups recovered after birth (postnatal demise); and 3) mice surviving until postnatal day 21 (post-weaning). Genotype and sex were analyzed via multiplex PCR amplification using the *Cite*-F/*Cite*-R primers targeting the *neomycin* cassette (present only in Ts1Cje mice) and *Sry*-F/*Sry*-R primers directed against the *Sry* gene (present only in males). For each reaction, *Fez*-F/*Fez*-R primers were used as endogenous controls. PCR conditions and amplification were as previously described [15]. Primer information and amplicon sizes are shown in the S1 Supplementary Methods.

### Embryonic and Postnatal Growth Analysis

To establish if there were growth differences between Ts1Cje mice and WT littermates, we obtained weights and crown-rump length (CRL) measurements at embryonic day 15.5 from 85 embryos, including 41 Ts1Cje and 44 WT littermates. Weights and total lengths were also recorded on a daily basis during postnatal life (starting at P3 and ending at weaning on P21) for a total of 96 pups (32 Ts1Cje and 64 WT). Pups were divided into two groups: Ts1Cje-I pups were defined as those that survived post-weaning and Ts1Cje-II pups died at some point before P21. A retrospective analysis of weights and lengths was performed for Ts1Cje-I (post-weaning survivors) and Ts1Cje-II (pre-weaning deceased) mice.

### Histological Examination

To assess for the presence of congenital heart defects in Ts1Cje embryos, pregnant C57Bl/6J female mice were sacrificed on E15.5, as described above. Fifty-one embryos were dissected and transferred into Bouin’s fixative solution for 24 h and rinsed twice in ethanol 50% for 2 min. Before paraffin embedding, samples were dehydrated in baths of increasing ethanol concentrations [70% for 24 h (3x8 h); 85% for 2 h (2x1 h); 90% for 2 h (2x1 h); 95% for 2 h (2x1 h) and 100% (3x40 min)], then cleared in xylene (3x20 min). Six-micrometer (μm) sections were obtained of each pup’s trunk using a rotary microtome (Leica Biosystems, Buffalo Grove, IL). For each embryo, at least 60 serial frontal or transverse sections of the heart were obtained and stained with hematoxylin and eosin (H&E) as described previously [21]. The presence of heart defects was analyzed using a light microscope (Axioskop, Zeiss, Thornwood, NY) by two different examiners blinded to genotype. After histologic analysis, the code was broken for genotype. One Ts1Cje embryo was excluded from analysis given sectioning problems that precluded assessment of the heart.

### Neonatal Behavioral Analysis

We used the modified version of the Fox scale as described by Hill *et al* [22] to investigate developmental milestones in Ts1Cje neonates (n = 32) versus WT littermates (n = 64). We compared the performances of Ts1Cje-II pups (pre-weaning deceased) (n = 16) that died before weaning with Ts1Cje-I (post-weaning survivors) (n = 16) and WT pups that survived after weaning. We used the surface righting, negative geotaxis, cliff aversion and forelimb grasp tests. Ts1Cje pups and their age-matched controls were evaluated on a daily basis starting at P3 and ending at P21. The experiments started on day P3 to avoid maternal stress and cannibalism during the first two days after birth, a phenomenon observed in our preliminary pilot experiments. The amount of time (latency) needed to complete each test was recorded and analyzed. Although we have previously reported on neonatal behavior in Ts1Cje mice [14], in our earlier experiments we did not recognize if postnatal growth patterns affected neonatal milestones. Therefore, in these experiments, we paid close attention to postnatal growth trajectories.

### Statistical Analysis

Mortality was inferred from the differences in the proportions of Ts1Cje and WT embryos, dead neonates, and post-weaning survivors. The Chi-square test was used to determine differences between groups. A *p* value of ≤ 0.05 was considered significant.

Before choosing the appropriate statistical tests to determine significant differences in weight, length and developmental milestones, the Agostino-Pearson Omnibus normality and the F tests were used to check whether the data followed a normal distribution and the variances were equal, respectively. Parametric Student and ANOVA tests were used if these two conditions were fulfilled. Otherwise, the non-parametric Mann-Whitney and Kruskal-Wallis tests were used. For multiple comparisons, Tukey’s and Dunn’s statistical test was performed with ANOVA and Kruskal-Wallis tests, respectively. A p value of <0.05 was used to define significance.

In order to help us understand the ability of early postnatal weight and body length to distinguish between Ts1Cje-II (pre-weaning deceased) versus Ts1Cje-I (post-weaning survivors) pups, we performed analyses of area under the ROC (Receiver Operator Characteristic) curve.

Graphs were plotted as mean ± SEM and all statistical analyses were performed using the GraphPad Prism 6.0 software package (GraphPad Software Inc., La Jolla, CA).

## Results

### Embryo and Neonatal Genotype and Sex Distribution in Ts1Cje Mice

**Fig 1:**
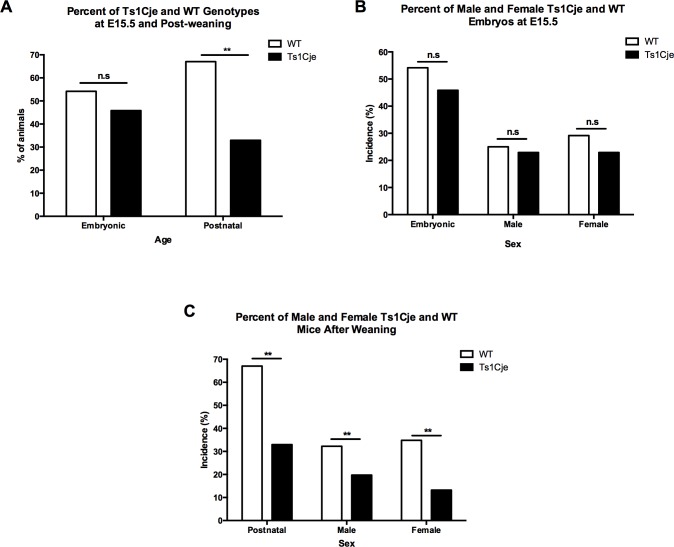
Prenatal and Postnatal Genotype and Sex Distribution in the Ts1Cje Mice Versus WT Littermates. A) Ts1Cje incidence at embryonic day E15.5 and postnatally. B) Ts1Cje sex distribution at embryonic day E15.5. C) Ts1Cje sex distribution during postnatal life.

A total of 144 embryos from 19 litters were recovered at E15.5. Among these embryos, 66 (45.8%) were Ts1Cje and 78 (54.2%) were WT (difference not statistically significant) (Table 1, Fig 1A). In pups that survived to weaning (n = 656, 131 litters), 28.2% (n = 185) were Ts1Cje, whereas the remaining 71.8% (n = 471) were WT [Chi^2^(1,n = 800) = 4.13, *p<0*.*001*] (Table 1, Fig 1A). In order to understand the influence of the genetic background, we generated a small colony of Ts1Cje on the B6EiC3Sn.BLiAF1/J background, and compared their prevalence versus their B6EiC3Sn.BLiAF1/J WT littermates. Out of 149 mice that survived after weaning, 66 mice (44.3%) were Ts1Cje and 83 (55.7%) were WT (Data not shown, p>0.05).

**Table 1:** Incidence of trisomy in Ts1Cje mouse model at different stages of life.

Parameter Analyzed	Embryonic (E15.5)	Postnatal Survivors at P21
**Non sex-stratified analysis**		
**Total number of embryos/pups analyzed**	144	656
**Total number of litters analyzed**	19	131
**Average number of embryos/pups per litter**	7.6	5.0
**Total number of WT embryos/pups**	78	471
**Total number of Ts1Cje embryos/pups**	66	185
**Percent Ts1Cje in utero/postnatally**	**45.8%**	**28.2%**
**Incidence of trisomy in males**		
**Total number of male embryos/pups**	69	327
**Percent of males**	47.9%	49.8%
**Total number of WT males**	36	220
**Percent of WT males**	25%	33.5%
**Total number of Ts1Cje males**	33	107
**Percent of Ts1Cje males**	**22.9%**	**16.3%**
**Incidence of trisomy in females**		
**Total number of female embryos/pups**	75	329
**Percent of females**	52.1%	50.1%
**Total number of WT females**	42	251
**Percent of WT females**	29.2%	38.3%
**Total number of Ts1Cje females**	33	78
**Percent of Ts1Cje females**	**22.9%**	**11.9%**

Sex ratios were also assessed in embryos and mice surviving post-weaning (Fig 1B and 1C). There was no predominance of one sex over the other in the Ts1Cje E15.5 embryos (33 males vs. 33 females) [Chi^2^(1,n = 144) = 0.212, *p = 0*.*645*] (Table 1, Fig 1B). However, analyses of sex ratios of post-weaning survivors showed a male predominance (107 vs. 78, ratio of 1.4:1) in the Ts1Cje mice, compared to a slight female predominance (251 vs. 220, ratio of 1.14:1) in the WT mice [Chi^2^(1,n = 656) = 5.28, *p* = *0*.*01*] (Table 1, Fig 1C).

### Prevalence of Heart Defects in Ts1Cje Mice

**Fig 2:**
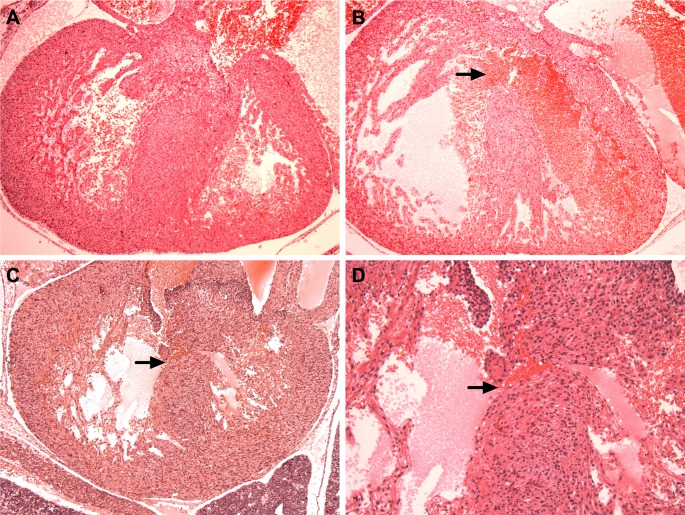
The Presence of Heart Defects in the Ts1Cje Mouse Model. Hematoxylin/eosin stained sections of WT (**A**) and two different Ts1Cje (B and C) embryonic day 15.5 hearts. (**A**) Normal ventricular septum in WT embryo (10X magnification); (**B**) First Ts1Cje embryo with a large ventricular septal defect or VSD (arrow, 10X magnification). (**C**) Second Ts1Cje embryo with a small ventricular septal defect (arrow, 10X magnification). (D) Higher magnification (20X) of the second Ts1Cje embryonic heart showing the small VSD and blood cells flowing between the right and left ventricles.

We analyzed a total of 29 Ts1Cje and 22 WT embryos for the presence of heart defects using hematoxylin/eosin staining. We only analyzed heart defects in E15.5 embryos and not in deceased pups in order to capture the incidence of heart defects in the Ts1Cje mouse model. We identified ventricular septal defects (VSDs) in 6/29 (21%) Ts1Cje embryos (Fig 2). In our cohort of Ts1Cje embryos and using our methodology, we were not able to identify atrio-ventricular septal defects (AVSD), atrial septal defects (ASD) or great vessel abnormalities. No septal defects or great vessel abnormalities were noted in the 22 WT littermates used as controls.

Given the small cohort, we did not have enough statistical power to assess for a correlation between low embryonic weight and crown-rump length and the incidence of heart defects in the Ts1Cje embryos.

### Growth Delays in in the Ts1Cje Mouse Model

The mean total body weight and crown-rump length (CRL) measurements between Ts1Cje (0.49±0.02 g and 16.11±0.17mm, respectively) and WT (0.46±0.02 g and 16.22±0.24mm, respectively) embryos were not significantly different (*p* = 0.24 for weight and *p* = 0.70 for CRL, unpaired t-test) (Data not shown).

**Fig 3:**
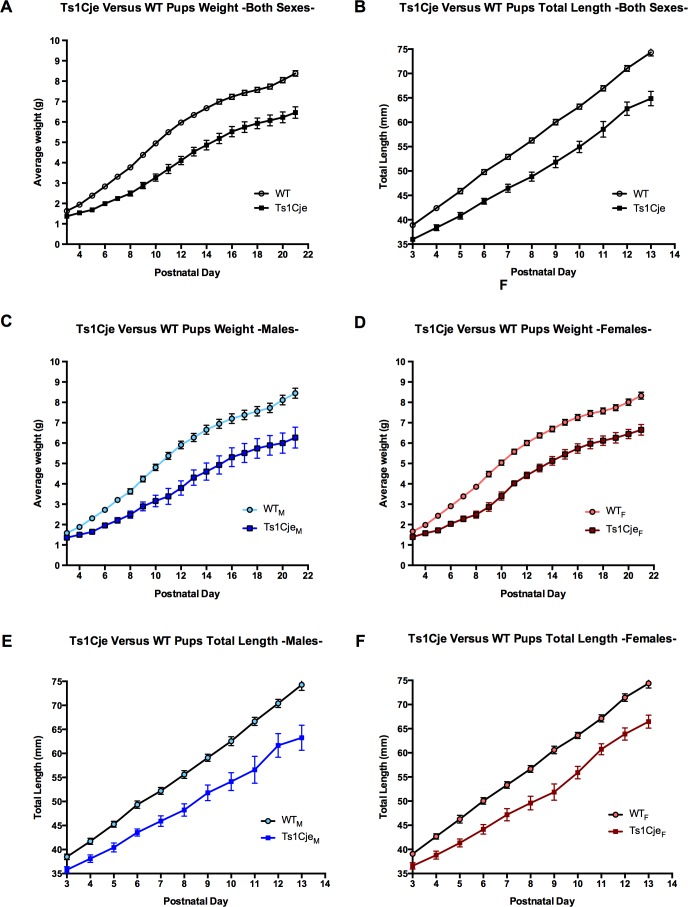
Pre-weaning growth profile of the Ts1Cje and WT neonates. Body weight and length were measured on a daily basis and compared between Ts1Cje and WT littermates. Ts1Cje neonates were consistently lighter and smaller than WT neonates (**A-B**). Sex stratified analyses revealed that both Ts1Cje males and females showed growth delays compared to their sex-matched WT littermates (**C-E**). Data are represented as mean ± SEM.

Total body weight and length measurements were also recorded postnatally on a daily basis from P3 through P21 (time of weaning). Ts1Cje mice (n = 32) showed delayed growth throughout the entire postnatal period, compared to their WT littermates (n = 64) (body weight = 1.34±0.04mm and 1.63±0.03 g, respectively; body length = 36.07±0.44 g and 38.91±0.36mm, respectively, *p<0*.*0001*). Both male and female Ts1Cje mice showed growth delays during the pre-weaning period (Fig 3).

### Growth Restriction and Mortality Incidence in the Ts1Cje Mouse Model

**Fig 4:**
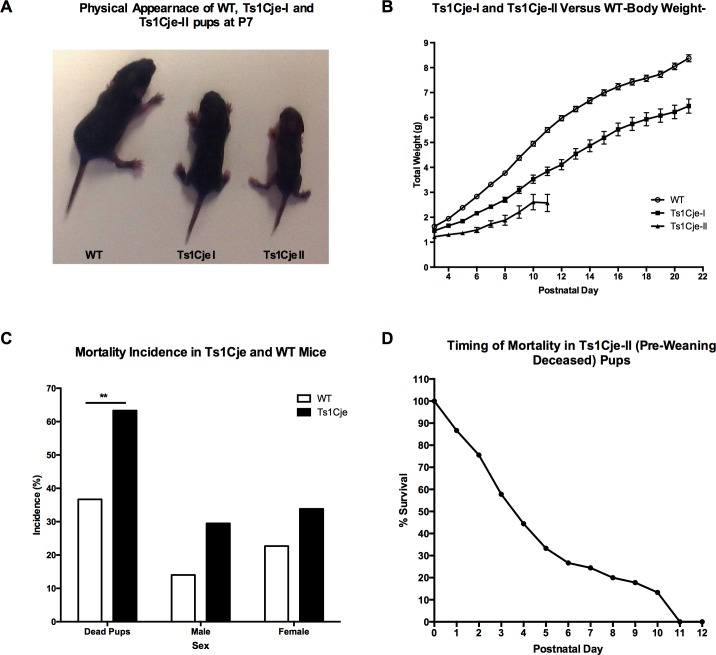
Growth Restriction and Postnatal Mortality in the Ts1Cje Mice Versus WT Littermates. A) Identification of two major growth phenotypes Ts1Cje-I and Ts1Cje-II: Representative picture of a WT pup (right), Ts1Cje-I pup (post-weaning survivors) (middle) and Ts1Cje-II (pre-weaning deceased) growth restricted pup at postnatal day 7 (left). B) Postnatal growth profiles of the Ts1Cje-I (post-weaning survivors) and Ts1Cje-II (pre-weaning deceased) mice. C) Postnatal mortality in the Ts1Cje mouse pups compared to WT littermates. D) Survival curve of the Ts1Cje-II (pre-weaning deceased) pups showing the percent of animals remaining at each postnatal day. Over 70% of the Ts1Cje-II (pre-weaning deceased) pups died by the end of the first week after birth and all Ts1Cje-II (pre-weaning deceased) pups died by postnatal day 11. Data are represented as mean ± SEM.

Among the Ts1Cje pups, we observed that there appeared to be two different Ts1Cje phenotypes characterized by significant growth and maturation differences. Using growth profile and postnatal demise as objective measures, we retrospectively designated them as Ts1Cje-I (post-weaning survivors) for the milder phenotype and Ts1Cje-II (pre-weaning deceased) for those that displayed severe growth delays and died at some time before P21 (Fig 4A). Statistical analyses demonstrated that weight and length differences were significant between wild-type, Ts1Cje-I (post-weaning survivors), and Ts1Cje-II (pre-weaning deceased) at all time points analyzed (F = 14.39 for weight and 12.26 for length, *p<0*.*0001*, One-way ANOVA) (Fig 4B). Tukey’s multiple comparison analysis showed that the growth profile of Ts1Cje-II pups (pre-weaning deceased) was significantly delayed compared to WT (*q = 6*.*98*, *p<0*.*0001*) and Ts1Cje-I pups (*q = 4*.*36*, *p<0*.*01*).

In order to further assess mortality in the Ts1Cje mouse model, we analyzed a total number of 207 dead neonates. Unfortunately, we were unable to recover 100% of the dead pups, because some pups were cannibalized by the dams during the dark phase. A higher proportion of the recovered dead pups were Ts1Cje (63.30%) and around a third (36.7%) were WT (Fig 4C). Mortality was slightly higher in females compared to males in both genotypes, but this increase was not statistically significant [Chi^2^(1,n = 112) = 1.383, *p = 0*.*24*)] (Table 2, Fig 4C). Postnatal mortality was primarily observed in the first two weeks after birth. We were able to record the exact postnatal day of death for 44 deceased Ts1Cje pups and found that over 70% of the Ts1Cje-II (pre-weaning deceased) pups died during the first postnatal week (Fig 4D).

**Table 2:** Early Postnatal Mortality Prevalence in Ts1Cje Mouse Model.

Group Analyzed	Total	Males (M)	Females (F)	Sex Ratio (F/M)
**Number of dead WT pups**	76	29	47	**1.62**
**% of dead WT pups**	**36.70**	**14.01**	**22.70**	
**Number of dead Ts1Cje pups**	131	61	70	**1.15**
**% of dead Ts1Cje pups**	**63.30**	**29.49**	**33.82**	
**Genotype Ratio (Ts1Cje/WT)**	**1.72**	**2.10**	**1.49**

**Fig 5:**
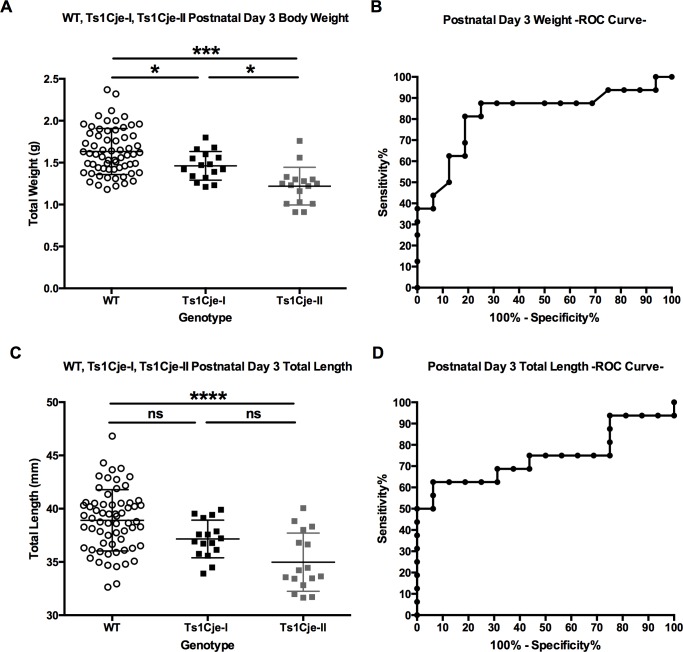
Growth Restriction Predicts Poor Postnatal Survival in the Ts1Cje Mouse Model. **A,C)** Body weight and length at postnatal day 3 of WT (n = 64), Ts1Cje-I (post-weaning survivors, n = 16) and Ts1Cje-II (pre-weaning deceased, n = 16). **B,D):** Receiver Operating Characteristic (ROC) curve representing the % sensitivity (the fraction of Ts1Cje pups that the test identifies as growth restricted) and % specificity (the fraction of Ts1Cje pups that the test identifies as non-growth restricted) at postnatal day 3 in the Ts1Cje mouse model. The ROC analysis showed that early postnatal body weight was a better predictor of postnatal survival in the Ts1Cje mouse model compared to body length (area under the curve = 0.82 for weight and 0.74 for length, *p* = *0*.*002* and *0*.*019*, respectively). Data are represented as mean ± SEM.

We successfully recorded the weights and lengths at P3 of 16 Ts1Cje-II (pre-weaning deceased) pups that died during the first two weeks of life, and compared them to the weights and lengths of their Ts1Cje-I (post-weaning survivors) (n = 16) and WT (n = 64) littermates that survived to weaning. Weights and lengths were significantly different between the three groups, with the Ts1Cje dead pups (Ts1Cje-II pre-weaning deceased) being significantly lighter and smaller (≈23% and 10% decreased, respectively) than the Ts1Cje-I (post-weaning survivors) and WT pups that survived after weaning (*p<0*.*001*, Kruskal-Wallis test) (Fig 5A and 5C). It is important to notice that the Ts1Cje-I (post-weaning survivors) and Ts1Cje-II (pre-weaning deceased) pups displayed a continuous spectrum of growth profiles with clear overlap between the two groups and measurable inter-individual variability (Fig 5A and 5C).

To investigate whether growth restriction (weight and length) could be used to determine the postnatal outcome (Ts1Cje-I post-weaning survivors or Ts1Cje-II pre-weaning deceased pups) in the Ts1Cje mouse model, we performed a Receiver-Operator characteristic (ROC) analysis of normalized weights and lengths of Ts1Cje-I (post-weaning survivors) and Ts1Cje-II (pre-weaning deceased) pups. Early postnatal body weight was a better predictor of postnatal survival in the Ts1Cje mouse model compared to body length (area under the curve = 0.82 for weight and 0.74 for length, *p* = *0*.*002* and *0*.*019*, respectively). (Fig 5B and 5D).

### Growth Restriction and Delayed Developmental Milestones in Ts1Cje Mice

**Fig 6:**
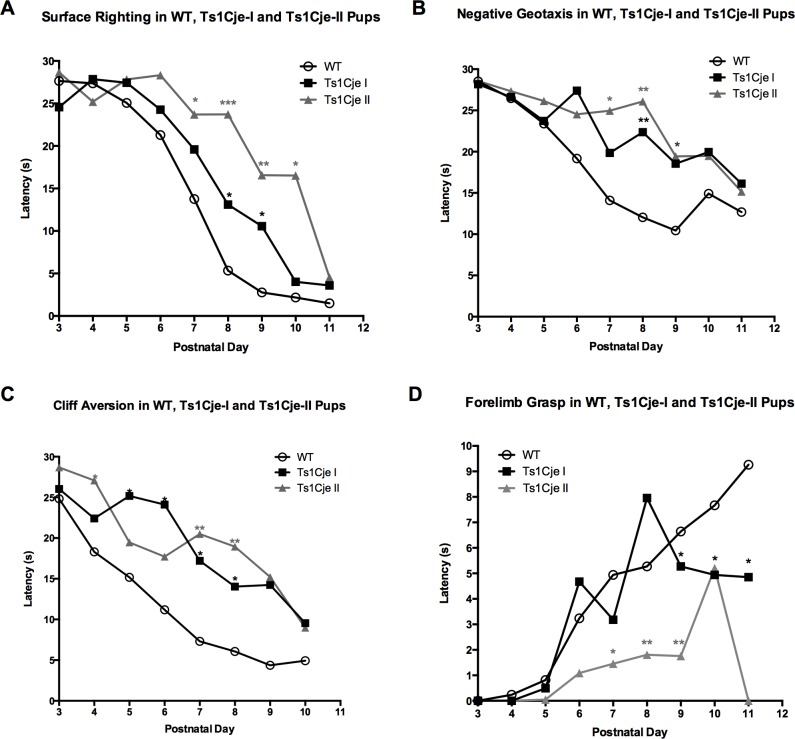
Developmental Milestones in the Ts1Cje-I (post-weaning survivors) and Ts1Cje-II (pre-weaning deceased) neonates versus WT littermates. Ts1Cje-II (pre-weaning deceased) growth restricted pups (n = 16) showed significant deficits in the surface righting (A), negative geotaxis (B), forelimb grasp (D) and, to a lesser extent, in the cliff aversion test (C) compared to the Ts1Cje-I (post-weaning survivors) (n = 16) and WT (n = 64) littermates. Data are represented as mean ± SEM. * (p<0.05), ** (p<0.01) and *** (p<0.001). Significance values presented in the figure represent comparisons to WT pups.

Analysis of the daily latency to perform the surface righting, negative geotaxis, cliff aversion and forelimb grasp tests revealed that the growth restricted Ts1Cje-II (pre-weaning deceased) pups performed significantly worse than the Ts1Cje-I (post-weaning survivors) and WT pups (Table 3, Fig 6).

**Table 3:** Developmental Milestones in WT, Ts1Cje-I (post-weaning survivors) and Ts1Cje-II (pre-weaning deceased) pups.

Developmental Milestone	Parameter Measured	WT (N = 61)	Ts1Cje-I (N = 16)	Ts1Cje-II (N = 16)
**Surface Righting**	**Latency delay significant at postnatal day versus WT**		P8*, P9*	P7*, P8***, P9**
	**Milestone Day**	7.85±0.16	8.81±0.49	9.00±0.59*
**Negative Geotaxis**	**Latency delay significant at postnatal day versus WT**		P8**, P9*	P7*, P8**
	**Milestone Day**	7.77±0.25	9.41±0.62*	10.67±0.62**
**Cliff Aversion**	**Latency delay significant at postnatal day versus WT**		P5*, P6*, P7*	P4*, P7**, P8**
	**Milestone Day**	6.42±0.22	9.12±0.49**	8.00±0.71*
**Forelimb Grasp**	**Latency delay significant at postnatal day versus WT**		P9*	P7*, P8**, P9**
	**Milestone Day**	7.46±0.14	8.00±0.52	9.86±0.63**

* (p<0.05)

** (p<0.01) and

*** (p<0.001).

Additionally, Ts1Cje-II (pre-weaning deceased) pups achieved the surface righting (9.00±0.59 days, *p<0*.*05*), negative geotaxis (10.67±0.62 days, p<0.01) and forelimb grasp (9.86±0.63 days, *p<0*.*01*) milestones significantly later than the Ts1Cje-I (post-weaning survivors) and WT pups (Table 3, S1 Fig).

## Discussion

In this study, we demonstrated the presence of neonatal, but not *in utero* mortality in the Ts1Cje mouse model of DS. We also showed sex differences in surviving mice and that this strain is associated with cardiac defects. The strengths of this study are its large size, the fact that the mice were studied with objective measures such as weight, mortality and neonatal milestones, and that both sexes were included.

### Ts1Cje Pre- and Postnatal Genotype and Sex Distribution

Our data demonstrated that roughly half of the embryos were Ts1Cje at E15.5, suggesting a Mendelian transmission of the Ts1Cje derivative chromosome. Similar transmission rates have been described for the Dp16 and the Ts65Dn models during fetal life [5, 9]. By the time of weaning, however, a non-Mendelian ratio was observed, with a decrease in prevalence of Ts1Cje pups to 28%. These results, as well as genetic analysis of the recovered dead pups, indicated a higher postnatal mortality in Ts1Cje pups. Comparable mortality prevalence (20–40%) has also been reported for the Ts65Dn and Dp16 weanlings [5, 9]. It is important to note that the true proportions of affected and WT mice are confounded by the fact that one can never analyze all of the lost pups due to maternal cannibalization. Based on data related to clinically recognized human pregnancies (spontaneous abortions, stillbirths and livebirths) and *in vitro* fertilization (IVF), Hassold and Hunt (2001) estimated the incidence of aneuploidy to be 5–25% with trisomies accounting for at least 50% of spontaneous abortions in humans [23]. Other human studies have reported a 24–45% difference between the incidence of DS observed at the time of second trimester amniocentesis and at full-term livebirths [24–28].

Here, we also showed that embryonic and postnatal genotype distributions of Ts1Cje mice differed by sex. While there was a 1:1 sex ratio in embryos, males predominated (59.9% vs. 40.1%) among Ts1Cje pups that survived to weaning. This is similar to the excess of males noted with most variants of human trisomy 21, except in mosaic cases [1, 27]. Male predominance in survivors has not been observed in the Ts65Dn mouse model [9]. Roper *et al* (2006) analyzed gender differences in the Ts65Dn model after weaning and found a non-significant reduction of trisomic males in weanlings compared to females (36% vs. 39%, *p* = 0.06). No analysis was performed to investigate the ratio of male to female embryos or among dead pups [9].

### Prevalence of Heart Defects in the Ts1Cje Mouse Model

In this study we found that 21% of Ts1Cje embryos had heart defects, suggesting that heart defects are a possible cause of postnatal mortality. Interestingly, we only observed VSDs. While our numbers were too small, these data are novel in the Ts1Cje mouse model. In our study, we did not identify heart defects in WT embryos. Heart defect; such as aortic arch (i.e. right aortic arch with an aberrant left subclavian artery) and atrioventricular and ventricular septal defects (ASDs, VSDs) have been reported in non-surviving Ts65Dn pups [17]. Raveau *et a*l (2012) showed that rescuing the gene dosage of the *App*-*Runx1* region in Ts65Dn mice improved electrocardiography and postnatal survival [29]. Cardiac malformations have also been observed in Dp(16)1Yey E18.5 embryos, including VSD, ASD, cleft mitral valves, severe coarctation of the aorta, double outlet right ventricle (DORV) and tetralogy of Fallot (TOF) [5]. In a recent study, Lana-Elola *et al* (2016) generated seven different mouse strains that carry different segmental trisomies of Hsa21 orthologous genes (including the Dp(16)Tyb1 mouse model equivalent to the Dp(16)1Yey model) to identify candidate genes or regions that are responsible for heart defects in DS [30]. They examined embryos at E14.5 using high-resolution episcopic microscopy (HREM) and found that 61.5% of the Dp1Tyb (carrying a duplication of 148 genes between *Lipi*-*Zbtb21* similar to the Dp16 mice) and 44% in the Dp3Tyb (carrying a duplication of 40 genes between *Mir802*-*Zbtb21*) present with heart defects, including AVSD, VSD and ASD. These authors, however, also identified a high incidence of heart defects in WT littermates from these two new strains (27% and 12% of WT in the Dp1Tyb and Dp3Tyb strains, respectively) [30].

### Growth Delays in the Ts1Cje Mouse Model and Humans with DS

Growth restriction has been associated with poor outcomes and increased lethality in human DS fetuses and infants [31–33]. In our study, significant growth differences were observed between the Ts1Cje and WT littermates during the first three weeks of life. In addition, we observed that postnatal growth delay was more likely to be associated with perinatal death within the first two weeks of life. Growth delays have also been reported in the Ts65Dn model, and can be detected as early as E9.5 (mid-gestation). To date, however, no correlations have been made between growth restriction and higher postnatal mortality in either Ts65Dn or Dp16 mice [5,9].

A correlation between growth restriction and neonatal mortality has also been observed in humans with DS. In a study that included 16,506 liveborn infants with DS, Kucik *et al* (2013) reported that low birth weight and congenital heart defects are the determining factors for poor survival in infants with DS. Infants with very low birth weight (<1,500 g) had 24 times the risk of dying compared with infants of normal birth weight (>2,500 g), and infants of low birth weight (1,500–2,499 g) had nearly 2.5-fold increase risk [33]. Similarly, Leonard *et al* (2000) reported on 440 live born infants with DS and showed that mortality was greater in females with DS and in those with low birth weights [34].

## Limitations of the Study

Although our study used large cohorts to establish the natural history of the Ts1Cje mouse model of DS, it had several limitations that need to be taken into account in future studies:

1. The true mortality incidence cannot be established given that we cannot recover all dead pups.
2. This incidence of heart defects incidence might be higher, as detection could be limited by our assessment technique. We were unable to identify heart defects other than VSDs using hematoxylin/eosin (H/E) staining. Combining H/E staining and high-resolution episcopic microscopy has the potential to detect more defects in mouse models of DS.
3. We did not record whether pups had a milk spot, so we don’t know if they failed to thrive because they were not feeding.
4. We did not assess the incidence of heart defects in the deceased pups and were unable to establish a direct correlation between growth restriction and presence of heart defects.
5. We did not assess for other congenital abnormalities such as oral clefts or gastrointestinal defects that could affect feeding behavior and have an impact on mortality.
6. We need more objective measures to better define phenotypic categories in the Ts1je mouse model and their relationship with cognitive outcome deficits.

## Conclusions and Future Studies

Here we provide the first detailed early natural history for fetal and neonatal Ts1Cje mice. Through this longitudinal analysis, we were able to show that the Ts1Cje derivative chromosome is prenatally transmitted according to Mendelian patterns of inheritance. Trisomic pups showed a higher incidence of early postnatal mortality associated with growth restriction and delayed achievement of neonatal milestones. We also provide the first histological evidence of heart defects in Ts1Cje embryos, which could be a potential cause of higher postnatal mortality rates.

To better understand the molecular mechanisms responsible for the presence of heart defects and growth restriction in a subset of animals, and that might predicts postnatal outcomes (mortality and cognition), future studies could include:

1. Genome wide association studies (GWAS) to identify single nucleotide polymorphisms (SNPs) that are associated with increased risk of heart defects.
2. The establishment of other objective endpoints (gene expression, mouse embryonic ultrasound imaging, high-resolution episcopic microscopy and histology) to define phenotypic categories in mouse models of DS.
3. The use of gene expression microarrays and proteomic techniques to identify pathway similarities and differences in tissue RNA and protein from Type I vs. Type II Ts1Cje.
4. Identification of prenatal and early neonatal therapeutic targets and molecules that can rescue these molecular and cellular abnormalities in mouse models of DS.

## Supporting Information

S1 Fig Neonatal Behavior Changes in the Fox Scale Tests in the Ts1Cje-I (post-weaning survivors) and Ts1Cje-II (pre-weaning deceased) Neonates. Delayed milestone day in the growth restricted Ts1Cje-II (pre-weaning deceased) pups compared to non-growth restricted Ts1Cje-I (post-weaning survivors) and WT neonates in the surface righting, negative geotaxis and forelimb grasp tests (**A, B** and **D**). Ts1Cje-I (post-weaning survivors) achieved the cliff aversion milestone at a later day than the Ts1Cj-II (pre-weaning deceased) and WT neonates (C). Data are represented as mean ± SEM.
(TIFF)

S1 Supplementary Methods Primer information and amplicon sizes for the primers used in Ts1Cje and sex genotyping. (DOCX)
