# Supplementary figures and images for: Perinatal Natural History of the Ts1Cje Mouse Model of Down Syndrome: Growth Restriction, Early Mortality, Heart Defects, and Delayed Development
Source: PLoS One. 2016 Dec 8;11(12):e0168009. doi: 10.1371/journal.pone.0168009 (PMC5145234; doi:10.1371/journal.pone.0168009)

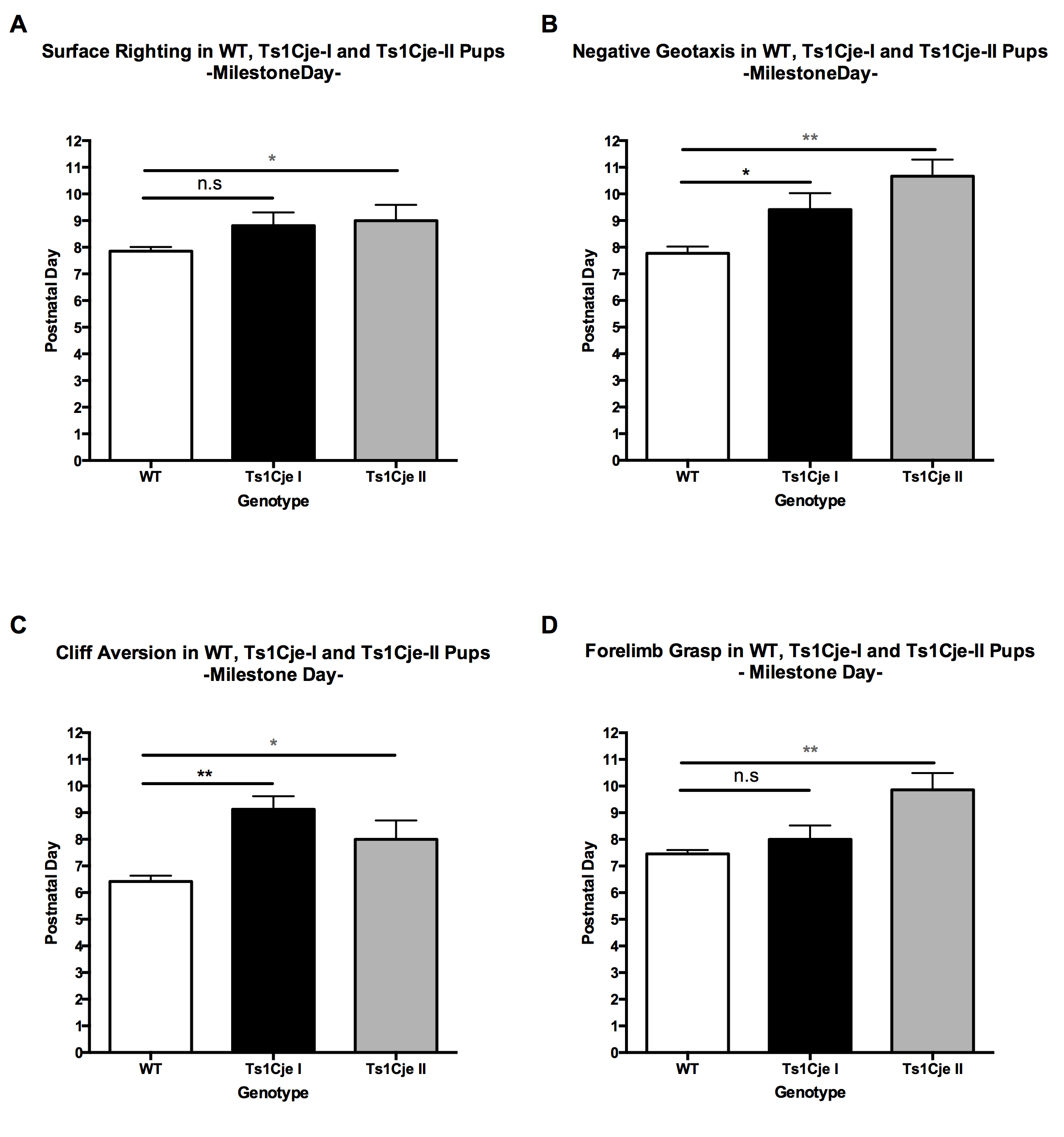

Supplement: S1 Fig — Delayed milestone day in the growth restricted Ts1Cje-II (pre-weaning deceased) pups compared to non-growth restricted Ts1Cje-I (post-weaning survivors) and WT neonates in the surface righting, negative geotaxis and forelimb grasp tests (A, B and D). Ts1Cje-I (post-weaning survivors) achieved the cliff aversion milestone at a later day than the Ts1Cj-II (pre-weaning deceased) and WT neonates (C). Data are represented as mean ± SEM. (TIFF) [file pone.0168009.s001.tiff]
