# Supplementary material for: Perinatal Natural History of the Ts1Cje Mouse Model of Down Syndrome: Growth Restriction, Early Mortality, Heart Defects, and Delayed Development
Source: PLoS One. 2016 Dec 8;11(12):e0168009. doi: 10.1371/journal.pone.0168009 (PMC5145234; doi:10.1371/journal.pone.0168009)
Supplement: S1 Supplementary Methods — (DOCX) [file pone.0168009.s002.docx]

**Full title: Perinatal Natural History of the Ts1Cje Mouse Model of Down Syndrome: Growth Restriction, Mortality, Heart Defects, and Delayed Development**

**Short title: Detailed Natural History of the Ts1Cje Mouse Model of Down Syndrome**

Millie A. Ferrés^1,2^, Diana W. Bianchi^1^, Ashley E. Siegel^1^, Roderick T. Bronson^3^, Gordon S. Huggins^4^, Faycal Guedj^1^

Supplementary Methods

**Supplementary Table I: Primer information and amplicon sizes for the primers used in Ts1Cje and sex genotyping**

| **Primer Name** | **Size** | **Sequence From 5’ to 3’** | **Amplicon Size (bp)** | **Target Gene** |
| --- | --- | --- | --- | --- |
| **Cite-19UP** | **25** | CTCGCCAAAGGAATGCAAGGTCTGT | **270** | **Neomycin cassette**  **(Ts1Cje)** |
| **Cite-324L** | **25** | CCCTTGTTGAATACGCTTGAGGAGA |  |  |
| **Sry-F** | **20** | TGATGGCATGTGGGTTCCTG | **221** | **Sry gene**  **(Males)** |
| **Sry-R** | **20** | GCTGGGATGCAGGTGGAAAA |  |  |
| **Fez-F** | **20** | AAGATCTGAGGCTCGCCAAG | **186** | **Endogenous control**  **(All)** |
| **Fez-R** | **20** | CTTCGGGAGCAGGTACCCTA |  |  |
